# Lumbago
1Read before the Bath and Bristol Branch of the British Medical Association, April 26th, 1905.


**Published:** 1906-06

**Authors:** Charles E. S. Flemming


### LUMBAGO.[1]

BY

CHARLES E. S. FLEMMING, M.R.C.S., L.R.C.P.

LUMBAGO is a painful immobilising affection of the muscles and fasciæ of the lumbar region, not of necessity accompanied by any marked or definite constitutional disturbance further than some lassitude and malaise. The onset of a first attack is very sudden, often occurring immediately or soon after a violent or quick movement of the muscles affected, at other times first noticed in moving from a position of rest. There is no warning of the first attack, but recurrences may be preceded for a day or two by some stiffness and slight aching of the parts. The pain is felt on any attempted active movement of the lower half of the trunk, especially on rotation or extension; it seems to spread from the spines of the lower lumbar vertebræ outwards on one or each side, along the crest of the ilium, upwards to the ribs and downwards to the sacral and gluteal regions. It is intense, and shooting or tearing in character, and on account of the way in which it spreads over the whole affected area it has been well called "sheet-lightning" pain. During rest there is usually no pain, more than a dull aching. There is considerable tenderness on one or both sides, either over the body of the erector spinæ or else over the attachments of this muscle and the quadratus lumborum to the sacrum and ilium; voluntary

[1] Read before the Bath and Bristol Branch of the British Medical Association, April 26th, 1905.

or active movement of these muscles is difficult or impossible. This acute stage lasts for from two to four or five days, is followed by much aching and stiffness for ten days or a fortnight, in some cases for a much longer period. This is an affection of adult age, much more frequent after forty than before.

Arranged in order of frequency, the erector spinæ, multifidus spinæ, quadratus lumborum, and latissimus dorsi are the muscles mostly affected, but their aponeuroses and the dense and inelastic deep fascia that overlies them are involved; possibly the sacro-iliac ligaments should be included in this list. Through these muscles or their aponeuroses pass the posterior branches of the gluteal and sacral nerves, some of which supply the skin over the gluteal region down as far as the trochanter major. The external branches of the three upper posterior sacral nerves pass through the multifidus spinæ to the outer surface of the great sacro-sciatic ligament, close to the great sciatic nerve.

The fasciæ, the aponeuroses, the muscular tissue proper, or the intra-muscular connective tissue may each or all be affected. The pain is probably due to implication of nerves passing through these structures or to the toxic irritation of muscle nerve endings.

There is little direct evidence as to the exact nature of this affection, it may be a myalgia or true muscular rheumatism with a cell exudation in the fibrous tissue between the muscle bundles—an exudation which in time may become fibrous—involving large or small tracts of one or all the muscles, or it may be a simple serous or sero-fibrinous effusion into the muscles, or between the dense and inelastic fasciæ and the muscles and aponeuroses.

An analogous case may help to explain this condition. I attended a man who the day before I saw him had taken part in a bicycle race. He was not in racing condition, and after the race he stood still and got wetted by a shower of rain. That night he had great pain and stiffness in his right thigh. I found him hardly able to move his leg, and the front and outer side of the thigh were swollen and painful. The swelling was most evident at the middle third of the outer side of the thigh, and the next day, although there was no pyrexia, I thought there was free fluid in the swelling and made a small puncture. Only a few drops of blood-stained serum escaped at the time. I applied a carbolic fomentation, and in half an hour the pain had almost ceased and did not return. One would fairly assume that in this case there was serous effusion under the fascia lata, and although one would hesitate to draw a definite conclusion from one such case, there is more inclination to do so on realising how often complete relief is given to lumbago by simple puncture.

To obtain evidence on the point of serous effusion I have twice punctured cases of lumbago with a long hypodermic needle, but without any definite result. However, it would be quite a chance whether the serum would happen to be drawn into the needle, seeing that the depth of the effusion, if any, is so uncertain, and that it is probably not in the form of free fluid.

The causes of this inflammation are varied: traumatic, bacterial, and chemical. The traumatic cause is probably always, except in the evident occasion of a direct injury from outside, a sudden muscular effort, the effect being either the rupture of some muscle fibres, or some fibres of an aponeurosis at its bony attachment, followed by a serous effusion into the neighbouring parts; but more usually a strain with the same effect, as is often seen in other parts, for example in the forearm when a teno-synovitis follows some violent muscular effort, or where effusion into the knee-joint follows a wrench at football or a strain while bowling. These are all simple traumatic effusions or inflammations, which may be and often are aggravated by other causes, such as cold, fatigue, rheumatism, or gout.

True rheumatic lumbago need not here occupy our attention, as it is merely a local manifestation of a definite specific disease, with the changes that attach to that disease in other muscles and fasciæ.

But I expect that the greater number of cases, though immediately caused by a toxin, are not the result of specific infection, but due to other interference with physiological processes. This may be either the local manifestation of a general toxæmia, or the toxic condition may be entirely local. As an example of the former, it is interesting to note the frequency of lumbar pain in such diseases as influenza and small-pox. The usual cause is the overloading of the blood with the products of muscular exertion and the toxins of fatigue, sarcolactic acid, creatinin, xanthine and a proteid toxin as yet undetermined. There may be an undue formation of these products, especially in those who are out of condition, or else there may be a deficient removal of these noxæ as the result of constipation, defective action of the kidneys, or inhibition of the secreting function of the skin, and just as the toxin of influenza causes lumbar pain, so may the products of fatigue cause lumbago; and it is evident that this condition will be intensified if the muscles in question have had extra and unusual work or have been strained, or if the tissues in the neighbourhood are exposed to sudden cold or great heat, or if anything occurs to interfere not only with the circulation of the blood, but with the circulation of the lymph, which in this position is possibly not as free as in the limbs. The presence of the noxa may irritate the nerve endings in the muscles and so cause the acute pain, but more probably it will cause a serous or sero-fibrinous effusion into the connective tissue of the muscles, the aponeuroses and the deep fascia.

While all pain in the lower half of the back is commonly called lumbago, it is not to be wondered at that there is some confusion as to the exact nature of this affection, so that it will be well to limit the term to those cases in which the pain is strictly in the lumbar region, occurs only on movement, and is accompanied by more or less definite tenderness. Though I say that the pain must be in the lumbar region, it may through the posterior lumbar and sacral nerves be referred to the gluteal region and the upper part of the back of the thigh, it may also spread by the quadratus lumborum and the latissimus dorsi upwards and outwards to the lower ribs and the hypochondriac region.

The great group of cases from which it is quite important to differentiate lumbago is that including affections of the pelvic organs where pain is referred to the lumbar region, and the symptom that may chiefly guide us is that the pain of pelvic disease is not increased simply by the movement of the lumbar muscles; it may be increased by standing or by some change of position, but the mere movement of these muscles while the patient is lying down does not increase the pain. We must be on the look-out for those numerous diseases which may produce a superficial semblance, and refuse to call any case lumbago until they have all been eliminated. The diseases which are perhaps the commonest causes of lumbar pain are piles, constipation and rectal catarrh, uterine displacement and endometritis, prostatic and bladder disease; but almost any pelvic disease may be included in this list, as also may renal calculus and the passage from the kidneys of uric acid crystals or very acid urine. The pain of malignant disease of the sacrum or ilium is too constantly severe to be mistaken. Difficulty in diagnosis is more usual in chronic and recurrent attacks of lumbago, as here the onset and character of the pain are less marked. More difficult to differentiate are diseases of the sacro-iliac synchondrosis and of the vertebræ and their adnexa. Tubercular or syphilitic disease is usually accompanied by other signs, and acute osteo-myelitis would be accompanied with fever and the pain more constant. Spondylitis deformans is probably not infrequently mistaken for lumbago, and only the occurrence of arthritis in other parts would render a diagnosis certain. We must remember that through the external branches of the three upper posterior sacral nerves inflammation may spread to the great sciatic nerve and so cause sciatica, a not infrequent consequence of lumbago.

In the first stage of an acute attack our object is to relieve the pain and to arrest the progress of the pathological changes; in the later stages not only to relieve pain but to restore the mobility of the parts, and in patients liable to recurrences to prevent them. In the acute stage I am sure that in some cases nothing gives so great relief as acupuncture. I say "in some cases," because it is not so in all. The more acute the pain the more sudden its onset and the greater the local tenderness the more likely is this proceeding to give relief. If my suggestion as to the pathological condition often present—serous effusion beneath the dense fascia—is correct, then we can see why puncture gives relief. The needles should be inserted deeply, two inches or more, into the tender muscular masses at the sides of the spine, left in two or three minutes, and followed by a fomentation. Twenty minutes or half an hour afterwards the patient must get up and walk. This should be followed by deep massage night and morning, and care taken that bowels, kidneys and skin all act well. Many patients will not submit to this not very painful treatment. Then a dose of calomel should be given, large fomentations applied at once, and some diaphoretic and diuretic drug given hourly, but no drug in my experience gives so much relief to the acute pain as does aspirin, which I usually give in addition to the above in doses of fifteen grains every four hours. When the acute stage has passed much remains to be done, but it must be done by the patient, who should walk and use his affected muscles several times every day, not for too long at a time for fear of setting up fresh trouble. Good massage and quite hot baths will help. Bowels and kidneys must be kept acting well and the back kept warm but not in a sweat. Diet should be light and stimulants entirely prohibited. In cases where there is in the later stages aching during rest I have known the constant current give much relief, ten to twenty milliampères, anode on abdomen, kathode on aching part. Relapses may be guarded against by regular and plentiful exercise, moderate feeding, avoidance of constipation and of undue fatigue, and warm but not impervious clothing on the back, in fact everything to promote good general health and good muscular condition, and to avoid the accumulation of the products of fatigue.

